# Synthesis of Bosutinib from 3-Methoxy-4-hydroxybenzoic Acid

**DOI:** 10.3390/molecules15064261

**Published:** 2010-06-11

**Authors:** Xiao Jia Yin, Guan Hong Xu, Xu Sun, Yan Peng, Xing Ji, Ke Jiang, Fei Li

**Affiliations:** 1 School of Pharmacy, Nanjing Medical University, Nanjing 210029, China; E-Mail: yinxiaojia@163.com (X.J.Y.); 2 Nanjing Children’s hospital, Nanjing Medical University, Nanjing 210008, China

**Keywords:** bosutinib, protein kinase inhibitors, 3-methoxy-4-hydroxybenzoic acid

## Abstract

This paper reports a novel synthesis of bosutinib starting from 3-methoxy-4-hydroxybenzoic acid. The process starts with esterification of the starting material, followed by alkylation, nitration, reduction, cyclization, chlorination and two successive amination reactions. The intermediates and target molecule were characterized by ^1^H-NMR, ^13^C-NMR, MS and the purities of all the compounds were determined by HPLC.

## 1. Introduction

**Scheme 1:**
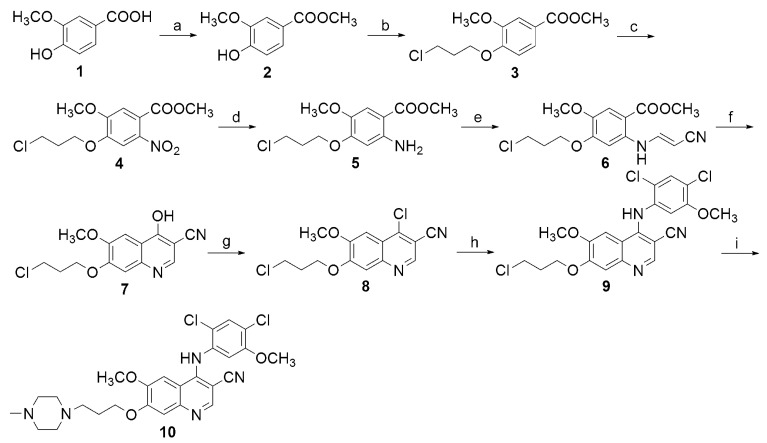
Novel synthesis of bosutinib.

The Src family of non-receptor protein tyrosine kinases (SFKs) plays key roles in regulating signal transduction, including cell growth, differentiation, cell shape, migration, and survival, and specialized cell signals [1]. However, c-Src was also identified as a proto-oncogene based on decades of research on an avian RNA tumor (sarcoma) virus. In some abnormal cases, such as mutation of the c-Src or over-expression, these enzymes can become hyperactivated, resulting in uncontrolled cell proliferation [2]. Bcr-Abl, the constitutively activated fusion protein product of the Philadelphia chromosome (Ph) is the principal oncogene underlying the pathology of chronic myelogenous leukemia (CML). Abl shares significant sequence homology with Src, and in its active conformation, bears remarkable structural resemblance with most SFKs. As a result, ATP-competitive compounds originally developed as Src inhibitors frequently exhibit potent inhibition of Abl kinase [3,4]. The second generation Bcr-Abl kinase inhibitors target both Src and Abl kinases to combat imatinib resistance, for example the Wyeth drug bosutinib (**10**, SKI-606, Scheme 1) has been evaluated for the treatment of CML in a Phase III clinical trial [5,6]. 

Several methods for the synthesis of bosutinib have been reported. Boschelli and Wang [5,7] started the route with protection of the hydroxy group of methyl 4-hydroxy-3-methoxybenzoate. The drawbacks are the facts that the cyclization reaction temperature (-78 ºC) is strict, and only gives a 47% yield. More important is that the overall yield is only 8.3%. Sutherland *et al.* [8] have designed a novel method for synthesizing bosutinib starting from 2-methoxy-5-nitrophenol. The overall yield was 32.1%, but the raw materials of the route are costly, and the reaction time is long, especially the last steps that required 66 h. 

In this report, we present a novel approach to synthesizing bosutinib. Compared with Boschelli’s route, it avoids the strict conditions of the cyclization reaction, and the overall yield is higher (21.7%). One the other hand, the starting materials are cheaper than Sutherland’s route.

## 2. Results and Discussion

Our novel synthesis of bosutinib (Scheme 1) started from 3-methoxy-4-hydroxybenzoic acid (**1**). This compound was esterified and then alkylated with 1-bromo-3-chloropropane to afford the intermediate **3** in 90.0% yield. Nitration of **3** with nitric acid in acetic acid gave compound **4**, which was reduced by powdered iron and ammonium chloride to give compound **5** in satisfactory yield (91.5%). In order to get a better reduction, Pd/C could be used to reduce compound **4** rather than Fe/NH_4_Cl. However, Pd/C gave incomplete conversions (85% yield) even after long reaction times (18 h). 

Compound **5** was reacted with 3,3-diethoxypropionitrile to obtain compound **6**. In this step, **5** was not directly reacted with 3,3-diethoxypropionitrile, but under the catalysis of trifluoroacetic acid, 3,3-diethoxypropionitrile was converted to 3-oxopropanenitrile, and then the 3-oxopropanenitrile was reacted with phenylamine **5** to form a Schiff’s base. Because of the p-π conjugation, the Schiff’s base would switch to another conformation **6**. 

Cyclization of **6** with sodium hydroxide and chlorination with POCl_3_ afforded compound **8**. The final product was obtained after two reactions with different amines. Compared with other methods, this new method is less costly because of the much cheaper starting materials used, consumes less time, and gives high yields. These results reported here provide the possibility of industrial production.

## 3. Experimental

### General

All reagents were purchased from commercial sources and used without further purification. Melting points were measured in open capillaries and are uncorrected. ^1^H-NMR spectra were recorded in CDCl_3_/DMSO-*d*_6_ on a Bruker Avance 300 spectrometer; chemical shifts (δ) are reported in parts per million (ppm) relative to tetramethylsilane (TMS), used as an internal standard. Mass spectra (MS) were obtained from Agilent 1100LC/MS Spectrometry Services. All compounds were routinely checked by TLC with silica gel GF-254 glass plates and viewed under UV light at 254 nm. The reported HPLC purity is the peak area calculated using Class-VP software on a Shimadzu 2010 instrument.

*Methyl 4-hydroxy-3-methoxybenzoate* (**2**). Thionyl chloride (30.0 g, 0.50 mol) was added dropwise at room temperature to a solution of 3-methoxy-4-hydroxybenzoic acid (**1**, 44.3 g, 0.26 mol) in methanol (500 mL). The mixture was stirred at room temperature for 2 h and the solvent was concentrated *in vacuo*. The oil formed was resolved in ice-water (50 mL), and pH was adjusted to 7-8 with saturated aqueous sodium bicarbonate solution. The solution was left standing in the refrigerator overnight, then the precipitate was collected by filtration, and air dried to give as brown power (49.0 g, 98% yield, 97.2% HPLC purity); ^1^H-NMR (CDCl_3_): 3.89 (s, 3H), 3.91 (s, 3H), 6.09 (s, 1H), 6.92 (d, *J* = 8.4 Hz, 1H), 7.55 (s, 1H), 7.62 (d, *J* = 8.3 Hz, 1H); ^13^C-NMR (CDCl_3_): 51.94, 56.08, 111.75, 114.07, 122.24, 124.17, 146.16, 150.12, 166.88; MS (ES) *m*/*z* 183.1 (M+1).

*Methyl 4-(3-chloropropoxy)-3-methoxybenzoate* (**3**). A mixture of methyl 4-hydroxy-3-methoxy- benzoate (**2**, 48.0 g, 0.26 mol), 1-bromo-3-chloropropane (50.0 g, 0.32 mol), and potassium carbonate (50.0 g, 0.36 mol) in DMF (125 mL) was heated at 70 ºC for 1 h. The reaction mixture was cooled to room temperature, and then poured slowly into ice-water (1.5 L) while stirring constantly. The solid formed was filtered off and washed with cold water. The off-white product was recrystallized from ethyl acetate (120 mL) to afford **3** (61.3 g, 90% yield, 99.3% HPLC purity); ^1^H-NMR (CDCl_3_): 2.29–2.33 (m, 2H), 3.77 (t, *J* = 6.2 Hz, 2H), 3.89 (s, 3H), 3.91 (s, 3H), 4.22 (t, *J* = 6.0 Hz, 2H), 6.89 (d, *J* = 8.5 Hz, 1H), 7.55 (d, *J* = 2.0 Hz, 1H), 7.67 (dd, *J* = 2.0 Hz, 1H); ^13^C-NMR (CDCl_3_): 32.02, 41.36, 51.93, 55.98, 65.41, 111.83, 112.46, 122.95, 124.43, 148.94, 152.16, 166.78; MS (ES) *m*/*z* 259.1 (M+1).

*Methyl 4-(3-chloropropoxy)-5-methoxy-2-nitrobenzoate* (**4**). Nitric acid (84.5 mL, 66%) was added dropwise at room temperature to a solution of methyl 4-(3-chloropropoxy)-3-methoxybenzoate (**3**, 51.6 g, 0.20 mol) in a mixture of acetic acid (150 mL). This mixture was stirred at 60 ºC for 3–4 h. Then the mixture was washed with ice-water (2 × 50 mL). The organic layer was washed with saturated sodium bicarbonate to neutrality. The oil formed was stirred till solidified and then collected by filtration to afford the product as light yellow solid (54.0 g, 89% yield, 98.7% HPLC purity); ^1^H-NMR (CDCl_3_): 2.28–2.42 (m, 2H), 3.77 (t, *J* = 6.2 Hz, 2H), 3.91 (s, 3H), 3.96 (s, 3H), 4.24 (t, *J* = 6.0 Hz, 2H), 7.08 (s, 1H), 7.49 (s, 1H); ^13^C-NMR (CDCl_3_): 31.82, 41.00, 53.21, 56.54, 66.03, 108.32, 111.02, 121.89, 141.08, 149.54, 152.84, 166.25; MS (ES) *m*/*z* 304.1 (M+1).

*Methyl 2-amino-4-(3-chloropropoxy)-5-methoxybenzoate* (**5**). Powdered iron (5.6 g, 0.10 mol) and ammonium chloride (8.4 g, 0.157 mol) were added to a mixture of methanol (70 mL) and water (30 mL). The resulting suspension was heated at reflux for 10 min, then a solution of methyl 4-(3-chloropropoxy)-5-methoxy-2-nitrobenzoate (**4**, 9.1 g, 0.03 mol) in heated methanol (100 mL) was added dropwise. The mixture was heated at reflux for 4 h. The catalyst was filtered, and the methanol was evaporated from the filtrate. The residue was air dried to afford the product as white solid (7.5 g, 91.5% yield, 98.2% HPLC purity); ^1^H-NMR (CDCl_3_): 2.26–2.34 (m, 2H), 3.73 (t, *J* = 6.2 Hz, 2H), 3.80 (s, 3H), 3.85 (s, 3H), 4.15 (t, *J* = 6.0 Hz, 2H), 5.58(s, 2H), 6.18 (s, 1H), 7.31 (s, 1H); ^13^C-NMR (CDCl_3_): 31.94, 41.43, 51.33, 56.70, 65.11, 100.58, 102.51, 113.65, 140.85, 146.97, 154.17, 168.10; MS (ES) *m*/*z* 274.1(M+1).

*(E)-Methyl 4-(3-chloropropoxy)-2-(2-cyanovinylamino)-5-methoxybenzoate* (**6**). 3,3-Diethoxy- propionitrile (2 mL, 13.34 mmol), trifluoroacetic acid (4 mL) and water (1 mL) were stirred for 6 h at 5–10 ºC under an atmosphere of N_2_, and then a solution of methyl 2-amino-4-(3-chloro- propoxy)-5-methoxybenzoate (**5**, 2.0 g, 7.32 mmol) in ethyl acetate (8 mL) was added. The mixture was stirred for 10 min. The solid formed was filtered off and air dried to afford the product as light yellow solid (2.0 g, 84.3% yield, 98.7% HPLC purity); ^1^H-NMR (CDCl_3_): 2.31–2.39 (m, 2H), 3.79 (t, *J* = 6.2 Hz 2H), 3.85 (s, 3H), 3.90 (s, 3H), 4.26 (t, *J* = 6.0 Hz, 2H), 4.68(d, *J* = 6.8 Hz, 2H), 6.58(s, 1H), 7.42 (s, 1H), 7.53 (t, *J* = 17.7 Hz, 1H), 10.55 (d, *J* = 6.5 Hz, 1H); ^13^C-NMR (CDCl_3_): 31.87, 41.15, 51.11, 56.37, 65.61, 71.96, 97.65, 105.26, 113.52, 117.41, 138.63, 142.95, 144.17, 153.94, 168.18; MS (ES) *m*/*z* 324.9 (M+1).

*7-(3-Chloropropoxy)-4-hydroxy-6-methoxyquinoline-3-carbonitrile* (**7**). A solution of (*E*)-methyl 4-(3-chloropropoxy)-2-(2-cyanovinylamino)-5-methoxybenzoate (**6**, 1.5 g, 4.36 mmol) in ethanol (20 mL), the pH was adjusted to 12–13 with sodium hydroxide. And then the solution was stirred at room temperature for 6 h, the solution was adjusted to nurture with water. The solid formed was filtered off and air dried to afford light yellow solid (1.16 g, 85.8% yield, 98.6% HPLC purity); ^1^H-NMR (CDCl_3_): 2.25–2.27 (m, 2H), 3.81 (t, *J* = 3.0 Hz, 2H), 3.88 (s, 3H), 4.20 (t, *J* = 3.7 Hz, 2H), 7.09(s, 1H), 7.48 (s, 1H), 8.59 (t, 1H), 12.53 (s, 1H); ^13^C-NMR (CDCl_3_): 31.56, 41.17, 56.03, 65.47, 93.78, 101.01, 104.89, 115.35, 119.99, 134.88, 144.49, 147.89, 152.14, 154.56; MS (ES) *m*/*z* 293.0 (M+1).

*4-Chloro-7-(3-chloropropoxy)-6-methoxyquinoline-3-carbonitrile* (**8**). A mixture of 7-(3-chloro- propoxy)-4-hydroxy-6-methoxyquinoline-3-carbonitrile (**7**, 1.0 g, 3.42 mmol) and phosphorus oxychloride (4.7 g, 30.10 mmol) in toluene (10 mL) was heated at reflux for 2 h. The solution was concentrated, and the pH was adjusted to 7 with saturated aqueous sodium bicarbonate. The resultant precipitate was collected by filtration to provide **8** (0.98 g, 92.4% yield, 98.1% HPLC purity); ^1^H-NMR (CDCl_3_): 2.36–2.44 (m, 2H), 3.81 (t, *J* = 6.2 Hz, 2H), 4.06 (s, 3H), 4.37 (t, *J* = 6.0 Hz, 2H), 7.42(s, 1H), 7.46 (s, 1H), 8.78 (s, 1H), 12.53 (d, s, 1H); ^13^C-NMR (CDCl_3_): 31.69, 41.13, 56.35, 65.62, 97.78, 102.06, 109.30, 113.57, 115.35, 142.96, 144.15, 148.19, 152.14, 154.64; MS (ES) *m*/*z* 311, 313 (M+1).

*7-(3-Chloropropoxy)-4-(2,4-dichloro-5-methoxyphenylamino)-6-methoxyquinoline-3-carbonitrile* (**9**). A mixture of 2,4-dichloro-5-methoxyaniline (0.54 g, 2.80 mmol), pyridine hydrochloride (0.276 g, 2.44 mmol), and 4-chloro-7-(3-chloropropoxy)-6-methoxyquinoline-3-carbonitrile (**8**, 0.80 g, 2.56 mmol) in 2-ethoxyethanol (10 mL) was heated at reflux for 2.5 h. The reaction mixture was partitioned between ethyl acetate and saturated aqueous sodium bicarbonate. The organic layer was washed with water, filtered, and concentrated *in vacuo* until a solid began to appear. This solid was collected by filtration to provide an off-white solid (0.68 g, 60.2% yield, 99.0% HPLC purity); ^1^H-NMR (DMSO-*d*_6_): 2.22–2.33 (m, 2H), 3.83 (t, *J* = 6.0 Hz, 2H), 3.86 (s, 3H), 3.95 (s, 3H), 4.29 (t, *J* = 6.0 Hz 2H), 7.34 (s, 1H), 7.37 (s, 1H), 7.75 (s, 1H), 7.85 (s, 1H), 8.42 (s, 1H), 9.64 (s, 1H); ^13^C-NMR (CDCl_3_): 31.75, 41.18, 55.52, 56.35, 65.62, 85.91, 102.06, 104.24, 109.30, 111.57, 116.57, 117.43, 132.34, 135.49, 144.92, 148.14, 150.16, 152.14, 154.64, 155.32 MS (ES) *m*/*z* 465.8, 467.8 (M+1).

*4-(2,4-Dichloro-5-methoxyphenylamino]-6-methoxy-7-[3-(4-methylpiperazin-1-yl)propoxy]quinoline-*3-carbonitrile (**10**). A mixture of 7-(3-chloropropoxy)-4-(2,4-dichloro-5-methoxyphenyl- amino)-6-methoxyquinoline-3-carbonitrile (**9**, 0.328 g, 0.7 mmol) and sodium iodide (0.11 g, 0.70 mmol) in *N*-methylpiperazine (4 mL) was heated at 80 ºC for 12 h. The reaction mixture was concentrated in vacuo and partitioned between ethyl acetate and saturated aqueous sodium bicarbonate. The organic layer was washed with brine, dried over sodium sulfate, filtered, and concentrated in vacuo. The residue was purified by column chromatography, eluting with 30% methanol in dichloromethane. The fractions containing product were collected and concentrated *in vacuo*. Diethyl ether was added to the residue, and the light pink solid was collected by filtration (0.28 g, 75% yield, 98.7% HPLC purity): m.p. 116–120 ºC; ^1^H-NMR (DMSO-*d*_6_): 1.92–1.97 (m, 2H), 2.15 (s, 3H), 2.32–2.46 (m, 10H), 3.84 (s, 3H), 3.93 (s, 3H), 4.19 (t, *J* = 6.3 Hz, 2H), 7.31 (br s, 2H), 7.43 (s, 1H), 7.64 (s, 1H), 8.52 (s, 1H), 9.51 (s, 1H); ^13^C-NMR (CDCl_3_): 25.96, 45.68, 52.67, 52.67, 54.24, 54.72, 54.72, 56.01, 60.71, 66.87, 89.10, 101.66, 101.66, 109.12, 113.95, 117.17, 122.99, 122.99, 128.27, 137.88, 146.15, 148.13, 148.51, 149.50, 150.43, 153.03; MS (ES) *m*/*z* 530.2, 532.2 (M+1).

## 4. Conclusions

A novel synthesis of bosutinib starting from 3-methoxy-4-hydroxybenzoic acid has been established. Compared with the existing methods, this new method is less costly because of the much cheaper starting materials used, consumes less time, and gives higher yields. These results reported here offer the possibility of industrial production.

## Figures and Tables

a) CH_3_OH; b) 1-bromo-3-chloropropane; c) HNO_3_; d) Fe/NH_4_Cl; e) 3,3-diethoxypropionitrile, trifluoroacetic acid; f) NaOH; g) POCl_3_; h) 2,4-dichloro-5-methoxyaniline, pyridine hydrochloride; i) NaI, *N*-methylpiperazine.
